# Enhanced Stability and Amplified Signal Output of Single-Wall Carbon Nanotube-Based NH_3_-Sensitive Electrode after Dual Plasma Treatment

**DOI:** 10.3390/nano10061026

**Published:** 2020-05-27

**Authors:** Joon Hyub Kim, Joon-Hyung Jin, Nam Ki Min

**Affiliations:** 1 Department of Nanomechatronics Engineering, Pusan University, Busan, 2 Busandaehak-ro 63 beon-gil, Geumjeong-gu, Busan 46241, Korea; kim4539@pusan.ac.kr; 2 Department of Chemical Engineering, Kyonggi University, 154-42 Gwanggyosna-ro Yeongtong-gu, Suwon 16227, Korea; 3 Department of Control and Instrumentation Engineering, Korea University, 2511 Sejong-ro, Sejong 30019, Korea

**Keywords:** dual plasma treatment, oxygen plasma, self-healing, single-walled carbon nanotube, surface functionalization

## Abstract

Pristine nanomaterials are normally prepared using finely controlled fabrication processes. Because no imperfect nanostructure remains, they cannot be used directly as electrode substrates of functional devices. This is because perfectly organized nanostructures or nanomaterials commonly require posttreatment to generate intentionally, the kinds of desirable defects inside or on their surfaces that enable effective functionalization. Plasma treatment is an easier, simpler and more widely used way (relative to other methods) to modify a variety of nanomaterials, although plasma-functionalized nano surfaces commonly have a short lifetime. We present herein a dual plasma treatment (DPT) that significantly enhances the degree and lifetime of plasma-induced surface functional groups on single-walled carbon nanotubes (SWCNTs). The DPT process consists of two individually optimized oxygen–plasma treatments. The DPT-modified SWCNT functioned as a sensing material for ammonia gas for more than a month. It also provided more than three times the degree of functionality for amplified signal output than with a single-plasma-treated SWCNT electrode.

## 1. Introduction

Nanomaterials, recent, but not brand-new, are frequently encountered materials that have been broadly investigated. Moreover, some of them have been commercially successful. The unique optical, electrical or mechanical properties of nanomaterials have driven researchers to develop a variety of nanomaterials. Carbonaceous nano powders, nanowires, nanotubes and nanosheets have been widely used in a variety of research fields including sensors, energy harvesting and storage devices [1,2,3,4,5,6,7]. However, an electrode substrate that is simply modified with a single nanomaterial or with a group of nanomaterials prepared in a defect-free manner, lacks functionality. Thus, such a substrate cannot be employed directly as the base material of a highly ordered electrochemical electrode. Indeed, posttreatment of nanomaterials commonly means using a process that artificially generates defects on the surface of the nanomaterial to add functionalization. There are two basic posttreatments to promote functionalization: chemical and physical, the latter of which includes plasma treatments [8,9,10,11,12,13,14].

Most chemical treatments provide the ends and edges of target nanomaterials with excellent functionalization but leave poor functionalization on the surface areas. This is partly due to inhomogeneous dispersion of the nanomaterial in the gaseous or liquid chemical solutions used for the functionalization [15]. The situation is much more serious with one-dimensional or two-dimensional nanomaterials than with zero-dimensional one. In general, end and edge areas of nanomaterials are energetically more active due to higher surface energy; thus, they attract more chemical functional groups than do the lower-energy surface regions. By contrast, the plasma treatments available for nano dots, wires and sheets provide nanomaterials of any shape with evenly distributed functional groups and produce no environmentally hazardous chemicals [16,17,18,19,20,21].

Scanning electrochemical microscopy data show activated carbon materials introduced to the surfaces [22,23]. In addition, surface functionalization using plasma treatment can be finely controlled by adjusting the plasma power and the plasma treatment time [2].

One of the major drawbacks with the use of plasma treatment is that a plasma-activated surface is not permanent and degrades with time [24]. Examples include deactivation of plasma-functionalized polymer surfaces. The polar chemical functional groups formed on the polymer surface by the oxygen plasma tend to penetrate deep inside the polymer network, leaving only the nonpolar groups on the surface [25,26,27]. Plasma-functionalized nanomaterials also exhibit surface aging-related degradation of functions.

A dual plasma treatment (DPT) could offer a way to diminish the aging effect. Here, we introduce two oxygen–plasma treatments in series for the modification of intrinsically hydrophobic SWCNT. The aim was to make them more hydrophilic and simultaneously, to retain surface hydrophilicity for a reasonably long period of time. The conditions of the two individually optimized plasma treatments were developed to minimize plasma-induced damage to the target nanomaterial. The first oxygen plasma treatment was carried out at 20 W for 20 s and the second treatment at 10 W for 10 s. This allowed additional introduction of oxygen-containing chemical functional groups through enhanced defect-generation on the CNT surface. Partially self-healed nanomaterials are vulnerable to corrosive oxygen plasma, so the latter treatment was less extensive than the former one [28,29,30]. As a result, the DPT-based sensing electrode created to monitor NH_3_ exhibited enhanced stability and an amplified output signal.

## 2. Materials and Methods

Sulfuric acid (98%), hydrochloric acid (50%), 1-ethyl-3-(3-dimethyllamino-propyl)carbodiimide, N-hydroxy-succinimide ester, dichlorobenzene (DCB), potassium chloride, potassium ferricyanide and potassium ferrocyanide were purchased from Sigma-Aldrich (St. Louis, MO, USA). SWCNTs produced by arc-discharge from Hanwha Nanotech Co. (Incheon, Korea) were sequentially purified with heat to remove carbon nano particles and amorphous carbon and then with acid to eliminate catalytic metals. Finally, the SWCNTs so obtained were composed of about 70 wt% SWCNTs and less than 30 wt% impurities containing graphite (<20 wt%) and metallic catalysts (<10 wt%).

The two plasma treatment steps were individually optimized. The two plasma treatment steps were individually optimized using home-made plasma equipment (see Supplementary Materials Figure S1). The plasma power and duration (time) of the first and second steps were 20 W for 20 s and 10 W for 10 s, respectively. The plasma-treated SWCNT was electrochemically analyzed using cyclic voltammetry (CV). The three-electrode electrochemical cell system used, was composed of an Ag/AgCl reference electrode, Pt-wire counter electrode, SWCNT working electrode and a potentiostat/galvanostat (Princeton Applied Research, AZ, USA). Scanning electron microscopy (SEM) (Hitachi, Ltd., Tokyo, Japan) and atomic force microscopy (AFM) (Park Systems, Suwon, Korea) were used to identify the distinctive morphologies of untreated, single plasma-treated (SPT) and dual plasma-treated (DPT) SWCNT films. Changes in the chemical composition of the various SWCNT films were investigated using X-ray photoelectron spectroscopy (XPS) (ThermoVG, MA, USA), Fourier transform infrared (FT-IR) spectroscopy (Bruker, MA, USA) and Raman spectroscopy (LabRAM, Horiba KOREA, Bucheon, Korea). Variation of the hydrophilicity of the SWCNT surface caused by the plasma treatment was investigated using water-drop contact-angle measurement (SEO Co., Suwon, Korea).

SWCNT solution suitable for spray coating to form a SWCNT network was prepared as follows. A portion (3 mg) of SWCNT powder was tip-sonicated in a 150 mL DCB solution for 20 min. Then, the SWCNT-dispersed solution was ultra-centrifuged at 20,000 rpm for 20 min at 4 °C. A homogeneous SWCNT-dispersed solution was obtained by separating the supernatant containing many other insoluble materials, from the SWCNT aggregates. A homogeneous SWCNT-dispersed solution was obtained by separating the supernatant from the SWCNT aggregates containing many other insoluble materials. A glass wafer with a 71-nm-thick spray coating of SWCNT film, experimentally confirmed thickness with small SWCNT film resistance change as the SWCNT thickness increases, was finally annealed on a hot plate at 190 °C for 2 h, and then diced into sections of 2 × 2 cm. (see Supplementary Materials Figure S2). The interdigitated Pd electrodes for ammonia sensing were formed by lifting off a patterned Pd layer that was thermally evaporated to form a layer on top of the SWCNT network. Unnecessary SWCNT film was eliminated by plasma etching. The dimensions of the NH_3_ sensor were 3 × 9.8 mm and its active sensing area was 9 mm^2^. The sensing of ammonia gas using the DPT-SWCNT electrode was studied using an LCR meter (3532–50 LCR Hitester, Hioki, Japan)-equipped laboratory chamber (see Supplementary Materials Figure S3).

## 3. Results and Discussion

**Figure 1:**
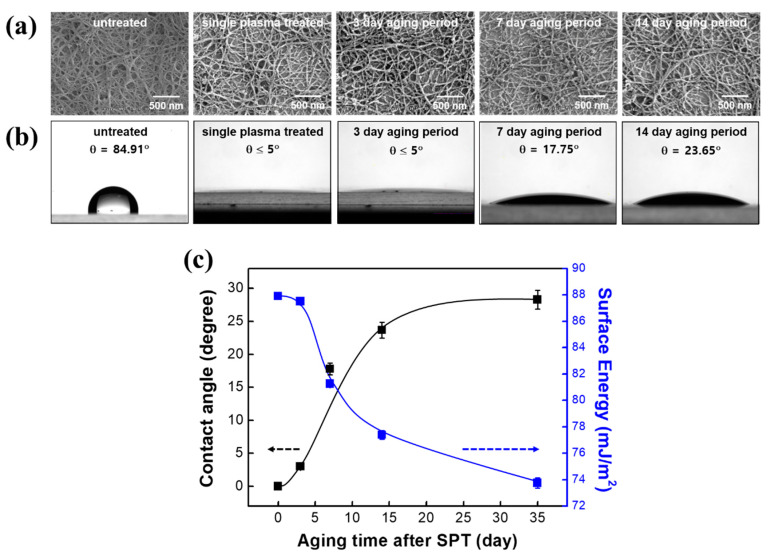
Changes in physical and responsive properties of SWCNT over time after the single plasma-treated (SPT) process. (**a**) SEM images; (**b**) water contact angles on the SPT-SWCNT surfaces for various aging times and (**c**) variations of the contact angle and the approximated surface energy as a function of atmospheric aging.

The sonopolymer produced in the process of vaporizing DCB to create a SWCNT network consists of C, H and Cl and a byproduct, such as sonopolymer, frequently observed on spray-coated SWCNT film [31]. Therefore, the outer surface of untreated SWCNT is generally contaminated by sonopolymer. The existence of the sonopolymer was confirmed by observing alkyl C–H stretching at 2800–2900 cm^−1^ using FT-IR spectroscopy (Supplementary Materials Figure S4). Generation of the peak related to the hydrogen-bonded hydroxyl groups and disappearance of the alkyl C-H stretching peak with the SPT-SWCNT film, indicate that sufficient defects were generated and that the sonopolymer was removed effectively from the surface. This was compatible with the reduced diameter of the CNT bundle after the plasma treatment (Figure 1a). The limited stability of the oxygen plasma-treated surface of nanomaterial is usually quantified using a water–drop contact–angle measurement. The thermodynamic work of adhesion (W_A_) represents how many chemical functional groups are formed on the plasma-treated surface and W_A_ and the water contact angle are related to each other by the Young–Dupré and extended Fowkes equations [32]. Figure 1b shows that the contact angle for the untreated SWCNT surface is about 84.91°. This high contact angle stems from the intrinsic hydrophobicity of the SWCNT and from the sonopolymer formed on the nano surfaces [33,34]. The SPT process decreased the angle to less than 5° due to hydrophilization of the nano surfaces and to the removal of organic residuals from the surface. While the surface morphology of the SPT-SWCNT was moderately altered every day, the water contact angle steeply increased to 24° in 14 days and was eventually saturated thereafter. Meanwhile, the surface energy, which was increased from 37 to 88 mJ m^−2^ by the SPT process, decreased and became saturated at around 74 mJ m^−2^ (Figure 1c). A fully optimized oxygen plasma process was employed in this work. This resulted in a high-quality SWCNT film with surface energy enhanced more than in a previous report [35].

Combination of the Young–Dupré and Owens–Wendt equations gives a linear relationship between the contact angle and the surface free energy. A dispersive component (γ_s_^d^) and polar component (γ_s_^p^) of the total solid surface energy (γ_s_) can be determined from the slope and the y-intercept of the combined equation (see Supplementary Materials, section on calculation of the surface free energy). The contribution for each component at various aging times is listed in Table 1. Before oxygen plasma treatment, most of the surface free energy of pristine SWCNT is attributable to the γ_s_^d^ component. The oxygen plasma substitutes polar functional groups for the surface carbon elements of the SWCNT with the result that the γ_s_^p^ component is mostly observed after the plasma treatment. While the γ_s_^d^ component increases, the γ_s_^p^ component decreases as the atmospheric aging time increases.

### 3.1. Electrochemical Characteristics of the DPT-SWCNT Electrode

**Figure 2:**
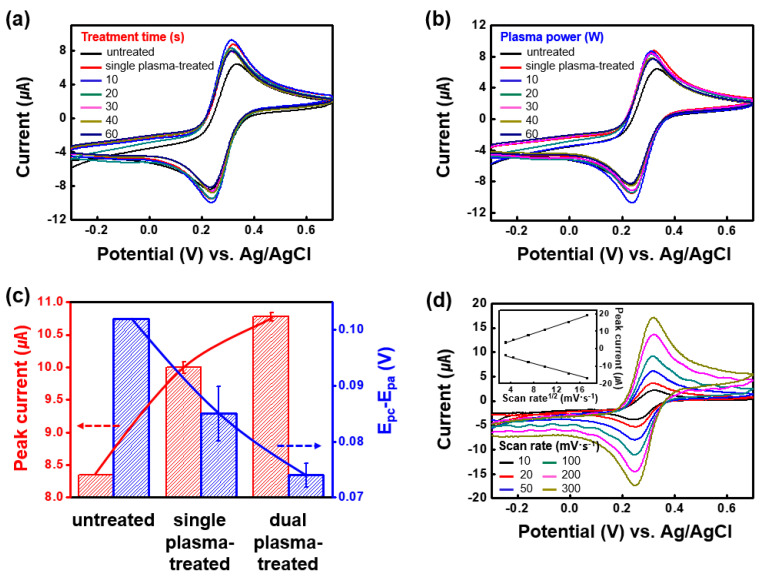
CV diagrams of (**a**) DPT-SWCNT electrodes prepared at various plasma treatment time (plasma power = 10 W) and (**b**) DPT-SWCNT electrodes prepared at various plasma treatment power (plasma treatment time = 10 s) in a 0.1-M KCl solution containing 10-mM Fe(CN)_6_^3−^/Fe(CN)_6_^4−^. Changes in the peak current and the peak potential separation of the untreated, SPT processed and DPT processed SWCNT electrodes are shown in (**c**); CV diagrams and Randles plot of the DPT-SWCNT electrode in the same electrolyte are shown in (**d**). Note that the CV curves of untreated SWCNT SPT-SWCNT are shown together in (**a**,**b**) for comparison.

The SWCNT electrodes were electrochemically characterized using CV in a 0.1-M KCl solution containing a 10-mM ferricyanide/ferrocyanide redox couple (Figure 2). The faradaic peak current increased from 8.35 µA (untreated SWCNT electrode) to 10 µA (SPT-SWCNT electrode), and then further to 10.8 µA with the DPT-SWCNT electrode. Simultaneously, the peak potential separation between the anodic and cathodic peaks (E_pc_—E_pa_) kept decreasing to 74 mV, which is close to the theoretical 59.2 mV in a one-electron-transfer-involved redox reaction. Constant peak potential separation in the range 10–300 mV∙s^−1^ (regardless of the scan rate applied) verified that the redox reaction occurred on the DPT-SWCNT electrode in a perfectly diffusion-controlled faradaic process. It also showed that the DPT process is ideal for preparing a SWCNT-based electrode with a fast electron transfer rate at the electrode–solution interface. The effective electrochemical area of the DPT-SWCNT electrode, as approximated by the Randles slope, was 0.04874 cm^2^, which is 5.13 times larger than the projected area. Detailed information about computation of the effective area using the Randles–Sevcik equation is available elsewhere [4,36].

### 3.2. Structural Analyses by Raman Spectra of the DPT-SWCNT Surface

**Figure 3:**
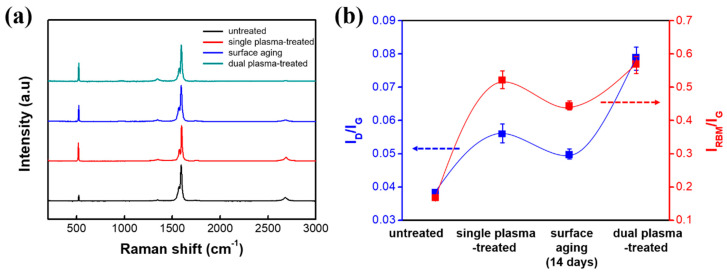
Changes in chemical structure and vibrational characteristics of the SPT- and the DPT-SWCNTs. (**a**) Raman spectra and (**b**) variations of I_D_/I_G_ and I_RBM_/I_G_ ratios.

Raman spectra of the SWCNT before and after the SPT and the DPT processes are shown in Figure 3a. The double peaks shown just below 1600 cm^−1^ (commonly called G-band) that are assigned to tangential C–C stretching vibrations, are still observed with the DPT-SWCNT. This indicates that the principal backbone of the nanotubes was preserved under all plasma conditions [37]. As can be seen in Figure 3b, oxygen plasma treatment provides effective defect generation on the surface of SWCNTs, enabling a high level of chemical functionalization without seriously damaging the nanostructured skeleton. In addition, the sonopolymer unintentionally coated onto the SWCNT surface during the preparation of the spray-coated SWCNT film prevents the untreated SWCNTs from free radial vibration. Furthermore, the decrease of the intensity ratio of the defects to the graphite (I_D_/I_G_) for aged SWCNTs means that SWCNTs have recovered naturally.

### 3.3. Composition Analyses by X-ray Photoelectron Spectra of the DPT-SWCNT Surface

**Figure 4:**
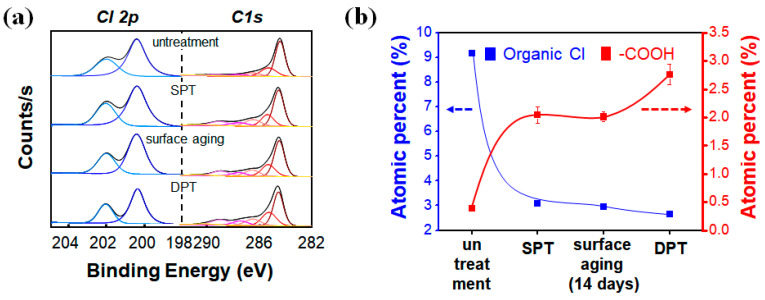
(**a**) Chloride 2p and carbon 1s peak shapes in the deconvoluted X-ray photoelectron spectroscopy (XPS) spectrum and (**b**) variations in atomic percent of the organic chloride and carboxyl groups.

Even though surface aging of the functionalized nanomaterial is unavoidable, the aging effect should be minimized to sustain the surface functionality for a reasonable duration. Multiple plasma treatments could provide a solution to such instability problems of plasma-activated nanomaterials. Enhanced stability and amplified signal output of the singe plasma-treated SWCNT film were observed after additional plasma processing of the SWCNT film. Changes in the composition of the four different SWCNT electrodes (i.e., untreated, SPT-, aged after SPT and DPT) were compared using their XPS spectra. The deconvoluted chloride 2p peak confirms the presence of sonopolymers (Figure 4a). The SPT process effectively removed the DCB residue, a contaminant of the spray coating process, which was verified by observing the diminished organic chloride (C–Cl) peak at 200.4 eV. This surface cleaning was almost completed by the SPT process and the decrease of the chloride 2p peak after the DPT process was negligible. The absorbance spectra of carbon 1 s for the SWCNT showed five characteristic peaks (Figure 4a). The strong peak at 285.01 ± 0.03 eV was assigned to sp2 hybridized and hydrogen-bonded carbon atoms, and that showing around 285.80 ± 0.12 eV was considered solely originating from sp2-hybridized carbon atoms. The other three peaks, located at 287.15 ± 0.21, 288.68 ± 0.34, 289.85 ± 0.18 eV and 291 ± 0.24 eV, were assigned to hydroxyl-group-bearing (C–O), carbonyl group (C=O), carboxyl group (–COO) and π-π*, respectively [38,39]. The DPT-SWCNT film had 2.8 times more carboxyl groups and the SWCNT film after the SPT process produced 1.2 times more carboxyl groups than the untreated SWCNT film did. The increased atomic percentage of the carboxyl group has great significance because the acidic carboxyl group is susceptible to easy formation of a thermodynamically stable amide bond by basic chemicals or amine groups of biomacromolecules under suitable catalytic conditions (Figure 4b). Table 2 summarizes the composition ratios of each of the components constituting the SWCNT.

### 3.4. Ammonia Gas Detection–Response Characteristics of the DPT-SWCNT Electrode

**Figure 5:**
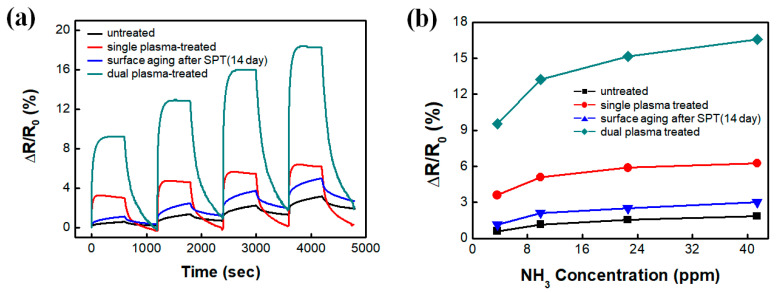
(**a**) Responses and (**b**) calibration curves of the SPT- and DPT-SWCNT sensors for monitoring NH_3_ gas.

The effectiveness of the DPT-functionalized SWCNT film as an electrochemical electrode was quantitatively evaluated by applying the DPT-functionalized film to monitor ammonia gas. The response time and sensitivity of the untreated SWCNT electrode were revised by the SPT process and further updated by the DPT process. The enhancement of its sensing performance is presumably due to the fact that the DPT process more effectively generates chemical functional groups on the nano surface than the SPT process does. Note that the DPT process actually treats SPT-SWCNT, while in the SPT process, the targets are untreated sonopolymer-contaminated nano surfaces [40,41]. The DPT-SWCNT electrode shows 2.5 times higher sensitivity and 3 times stronger output signal to NH_3_ gas than does the SPT-SWCNT electrode (Figure 5a). It also had a steeper sensitivity slope, especially in the concentration range when the gas is thin (Figure 5b). The stronger the output signal is, the better the signal-to-noise ratio because the real signal shows noise immunity. In addition, both the SPT- and DPT-SWCNT electrodes show response times shorter than 100 s.

### 3.5. Aging Pattern of the DPT-SWCNT Network Films

**Figure 6:**
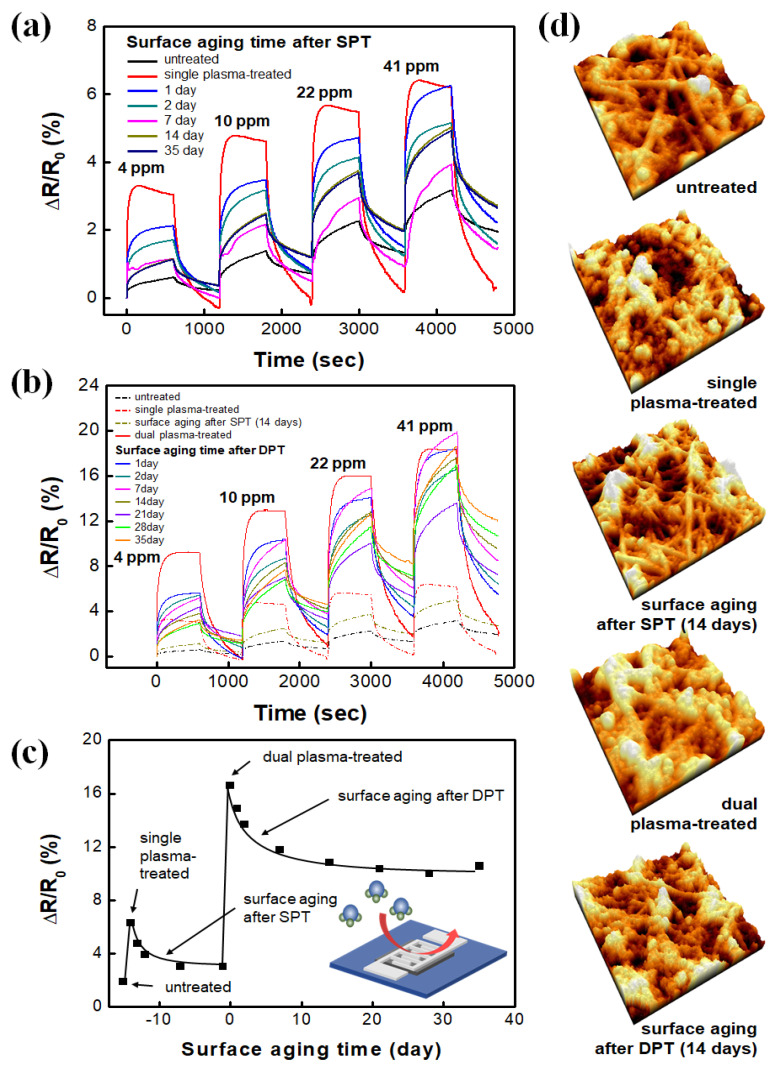
Dynamic responses to NH_3_ gas of variously aged (**a**) SPT-SWCNT and (**b**) DPT-SWCNT electrodes; (**c**) aging curves derived from responses to 41-ppm NH_3_ in (**a**,**b**,**d**) atomic force microscopy (AFM) surface images of the SWCNT films after each step of plasma treatment or 14-day-aging. Note that the variation in resistance to the NH_3_ concentration that increased about 3.3 times due to the plasma treatment decreased to 25% after two weeks. Note that purified N_2_ was used as a purging gas and to dilute the ammonia to the different experimental concentrations.

Time-dependent degradation in the performance of the SPT- and DPT-SWCNT sensitivity, which also causes change in the sensitivity of the SWCNT-based devices with time, was evaluated and compared. Figure 6a shows the degradation pattern of single plasma-treated (SPT) SWCNT-based ammonia detection in terms of gas sensitivity and response time. More detailed information about the optimized condition of the SPT process is available elsewhere [42]. Oxygen plasma enhances the sensitivity of the SWCNT ammonia sensor and reduces the response time to less than 100 s. Aging the plasma-treated SWCNT under atmospheric conditions decreases the sensitivity and increases the response time. Normalized resistance defined as the resistance variation (ΔR) divided by the initial resistance (R_0_), shows exponential decay with the aging time (Supplementary Materials Figure S5). Oxygen plasma creates C–O, C=O and O–C=O fractions during plasma-induced defect generation due to the bond dissociation of CNT-specific hexagonal alkyl chains. Those oxygen-containing polar fractions on the defect sites are more or less likely to form hydrogen bonds with electron-donating ammonia molecules. Surface aging causes loss of the polar fractions from the nano surfaces, which directly and simultaneously decreases the sensitivity and increases the sensor response time. Figure 6b shows changes of the response characteristics of the DPT-SWCNT electrode to NH_3_ gas as the aging time varies from 1 to 35 days. Decreased sensitivity and increased response time are observed with the DPT-SWCNT electrode (as with the SPT-SWCNT electrode. However, different time constants of the aging process (15 h for the SPT-SWCNT and 32 h for the DPT-SWCNT) verifies that the two SWCNT films age differently (Figure 6c). It is of great importance that the DPT-SWCNT saturates at a value 3.3 times higher than SPT-SWCNT does and therefore exhibits 3.3 times better functionality. Both the SPT and the DPT surfaces, which are shown as AFM images in Figure 6d, are both aged, but their degradation mechanisms seem to be different. As we have discussed, if the SPT process is used in a situation in which it should be aimed at surface cleaning, and it has a minor effect on the surface functionalization of the nanomaterials, nano surface functionalization by the SPT process will be limited. In the following step, a surface that was sufficiently cleaned in the previous step can be functionalized further, and more chemical functional groups are substituted. Two nearby functional groups (e.g., carboxylic acid groups) can be dimerized by hydrogen bonding between them to form a chemically more stable carboxylic acid dimer (see Supplementary Materials Figure S4 showing a strongly enhanced broad FT-IR peak of hydrogen-bonded hydroxyl groups formed by the DPT process). The dimerized functional groups are much more resistant to aerobic aging. Actually, the monomers and dimers of carboxylic acid form a thermodynamically equilibrated system.

## 4. Conclusions

In a plasma phase, each individually activated ion with the same electrical charge maintains a similar free-path distance from others, and the entire surface of a nanostructure eventually has the same opportunity to collide with the activated ions. This means that the whole surface area of the nanomaterial can be similarly functionalized by the plasma treatment with minimized unused area. In this work, two different, separately optimized oxygen plasma processes were developed, and they were employed to functionalize SWCNT. While the SPT process mainly played the role of removing surface contaminants from the nanomaterial, the major role of the DPT process, which requires two-week aging of the as-prepared SPT-SWCNT electrode as a prerequisite, was to chemically functionalize the nanomaterial. The different time constants of the SPT and DPT processed SWCNT probably means that the nano surfaces prepared using these two processes were governed by different aging mechanisms. We believe that the reason why the functionalized surface gradually loses its functionality is that the activated surface is self-healed and that oxygen-containing chemical functional groups are lost due to thermally or light-induced agitation at the surface. The meaning of self-healing, particularly in the CNT network, is that the partially damaged CNT-specific hexagon structure formed by the plasma treatment reforms into pentagon or heptagon structures with greater energy stability. While the defect generated CNT structure can be perfectly repaired at temperatures higher than 900 °C [43], an alternative partial healing process can occur at normal temperature and pressure that leaves plasma-induced defects remain to some extent. The findings in this work indicate that finely defined, multiple O_2_ plasma processes are desirable for the preparation of unique nanomaterials with enhanced peculiar functions that last longer than ever before.

## Figures and Tables

**Table 1:** Summary of the contributions of the γ_s_^p^ and γ_s_^p^ components to γ_s_ at various aging times.

Samples	$\gamma_{s}^{d}$ mJ m^−2^	$\gamma_{s}^{p}$ mJ m^−2^	$\gamma_{s}$ mJ m^−2^
Untreated	34.6	2.91	37.5
As-prepared (SPT)	1.96	85.9	87.9
3-day aging (SPT)	2.00	85.5	87.5
7-day aging (SPT)	2.80	78.5	81.3
14-day aging (SPT)	3.07	74.3	77.4
35-day aging (SPT)	3.34	70.4	73.7

**Table 2:** Variation in the composition of various SWCNTs confirmed by XPS spectra. Note that the optimized condition of the DPT process is effective only if the SPT-SWCNT electrode is aged under aerobic condition for 14 days.

	Organic Cl (%)	C sp^2^ (%)	C sp^3^ (%)	C–O (%)	C=O (%)	–COO (%)	π-π* (%)	COx/C (Total) (%)
Untreated	3.14	69.0	16.8	6.70	3.17	2.89	1.41	12.8
SPT (20W/20s)	0.47	57.9	18.6	10.3	5.14	6.68	1.37	22.1
Aged 14 days	0.42	57.8	18.8	9.79	5.54	6.59	1.55	21.9
DPT (10W/10s)	0.28	52.5	20.6	9.98	6.93	8.31	1.63	25.2

## Supplementary Materials

The following are available online at https://www.mdpi.com/2079-4991/10/6/1026/s1, Figure S1: Home-made plasma equipment used in this research. Figure S2: Plot of resistance as a function of SWCNT thickness. Figure S3: Schematic drawing of the LCR meter-equipped laboratory chamber for monitoring NH_3_ gas with the DPT-SWCNT electrode. Figure S4: FT-IR spectra of various SWCNT films. The spectra for untreated, the SPT-, surface aged and the DPT-SWCNT films are black, red, blue and dark cyan, respectively. The aliphatic C-H stretching vibration resulting from the sonolytic products of DCB was assigned to 2851 cm^−1^. This band decreased due to the removal of the sonopolymer by the SPT process. The broad–OH group shown around 3350m^−1^ was strongly enhanced by the DPT process presumably because the removal of the sonopolymer by the previous SPT process provided a better environment for the formation of carboxyl groups. Figure S5: ΔR/R_0_ of the SPT-SWCNT electrodes exposed to 41.4 ppm NH_3_ as a function of aging time.
