# Supplementary material for: Enhanced Stability and Amplified Signal Output of Single-Wall Carbon Nanotube-Based NH3-Sensitive Electrode after Dual Plasma Treatment
Source: Nanomaterials (Basel). 2020 May 27;10(6):1026. doi: 10.3390/nano10061026 (PMC7352858; doi:10.3390/nano10061026)
Supplement: Supplementary file 1 [file nanomaterials-10-01026-s001.pdf]

Enhanced Stability and Amplified Signal Output of Single-Wall Carbon Nanotube-Based NH_3-Sensitive Electrode after Dual Plasma Treatment

Joon Hyub Kim [1], Joon-Hyung Jin [2,*] and Nam Ki Min [3,*]

[1] Department of Nanomechatronics Engineering, Pusan University, Busan, 2 Busandaehak-ro 63 beon-gil, Geumjeong-gu, Busan 46241, Korea; kim4539@pusan.ac.kr

[2] Department of Chemical Engineering, Kyonggi University, 154-42 Gwanggyosna-ro Yeongtong-gu, Suwon 16227, Korea

[3] Department of Control and Instrumentation Engineering, Korea University, 2511 Sejong-ro, Sejong 30019, Korea

Abstract of Supplementary Materials

The Supplementary Materials for the primary manuscript entitled "Enhanced Stability and Amplified Signal Output of Single-Wall Carbon Nanotube-Based NH_3-Sensitive Electrode after Dual Plasma Treatment" includes information about an illustration of the LCR meter-equipped laboratory chamber, FT-IR spectra of various SWCNT films, calculation of the surface free energy, and response of the SPT-SWCNT electrodes to 41.4 ppm NH_3 over time.

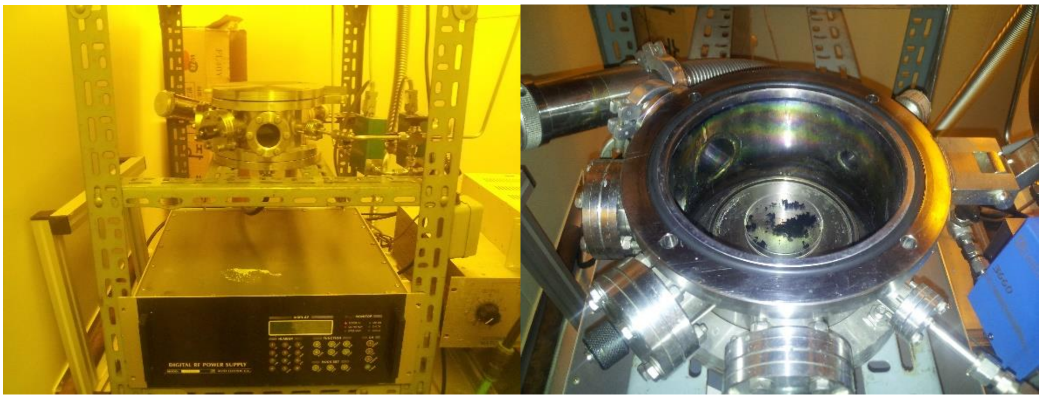

Figure S1. Home-made plasma equipment used in this research

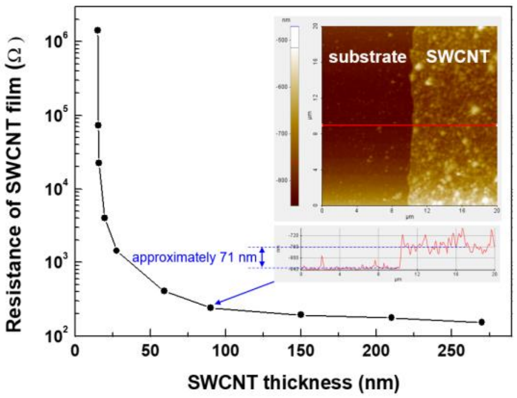

Figure S2. Plot of resistance as a function of SWCNT thickness. Inset: thickness measurement of used SWCNT through AFM

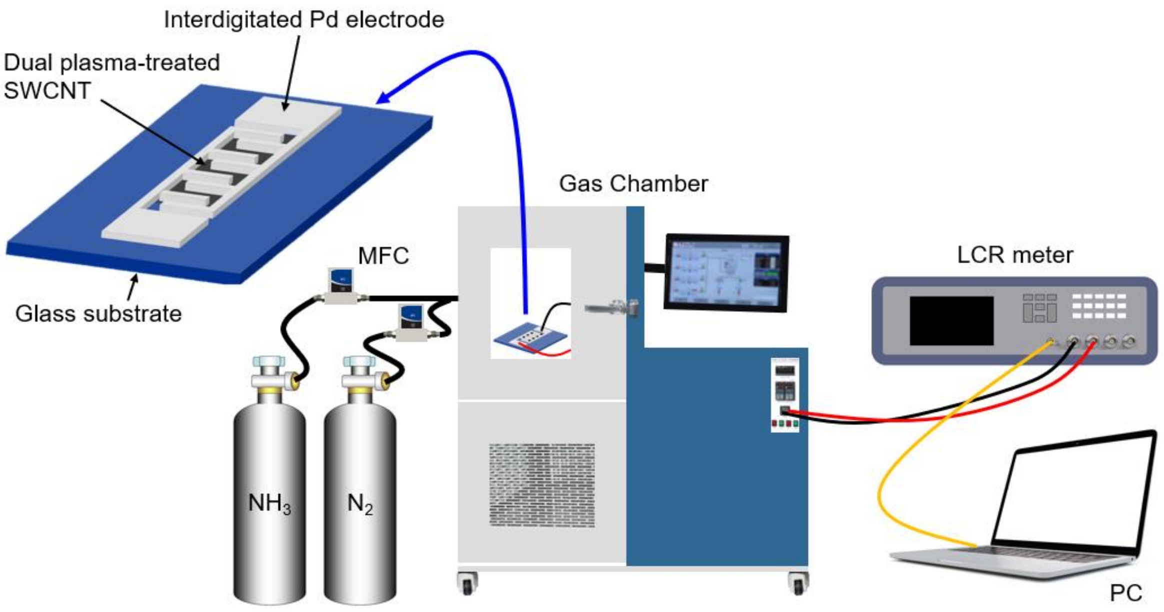

Figure S3. Schematic drawing of the LCR meter-equipped laboratory chamber for monitoring NH_3 gas with the DPT-SWCNT electrode.

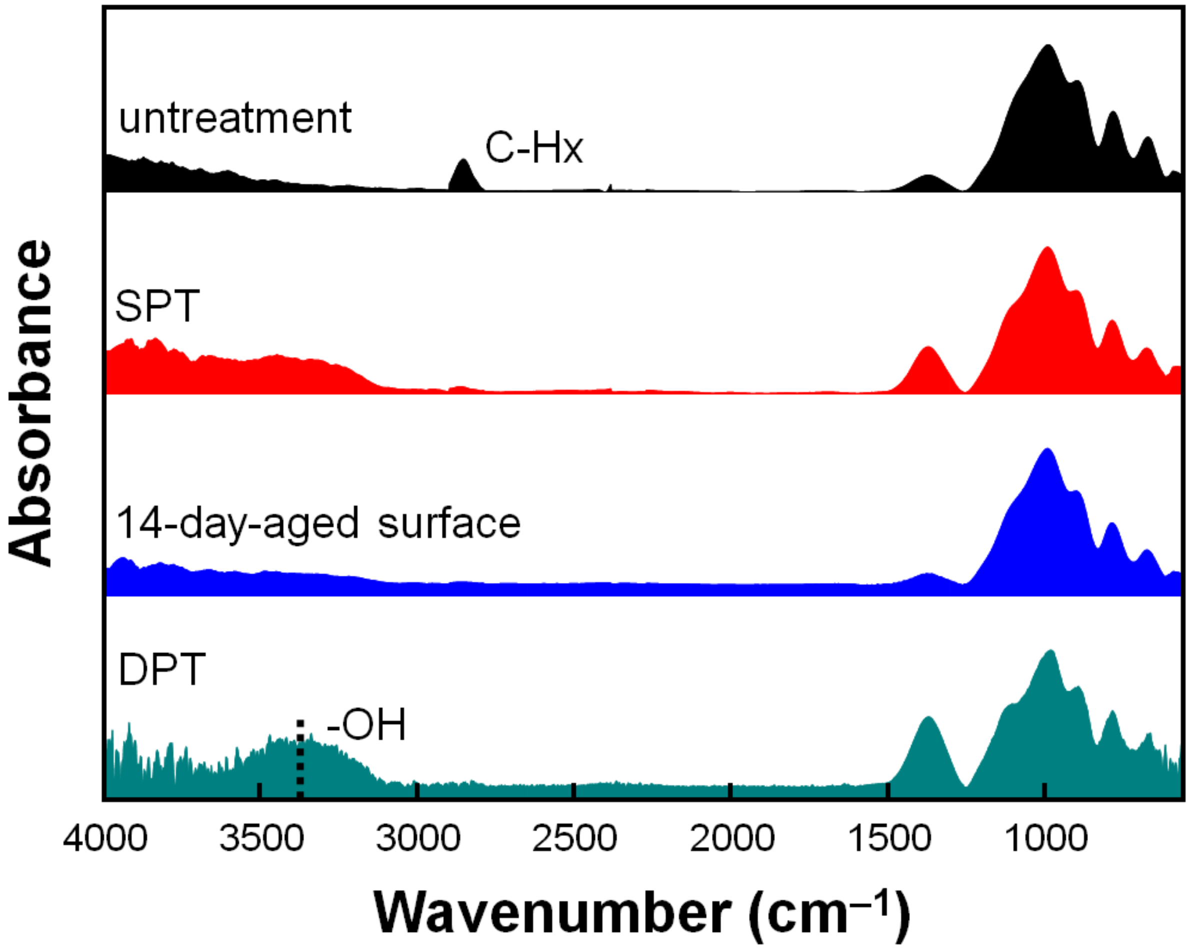

Figure S4. FT-IR spectra of various SWCNT films. The spectra for untreated, the SPT-, surface aged, and the DPT-SWCNT films are black, red, blue and dark cyan, respectively. The aliphatic C-H stretching vibration resulting from the sonolytic products of DCB was assigned to 2851 cm^{-1}. This band decreased due to the removal of the sonopolymer by the SPT process. The broad -OH group shown around 3350 cm^{-1} was strongly enhanced by the DPT process presumably because the removal of the sonopolymer by the previous SPT process provided a better environment for the formation of carboxyl groups.

Calculation of the surface free energy

Determination of the surface free energy of plasma-treated SWCNT-decorated solid is as follows.[1] The interfacial surface energy of a typical solid–liquid interface (γ_{sl}) according to Owens and Wendt equation is:[2]

$$\gamma_{sl} = \gamma_s + \gamma_l - 2\sqrt{\gamma_s^d \gamma_l^d} - 2\sqrt{\gamma_s^p \gamma_l^p} \quad (1)$$

where, surface energy of the solid (γ_s), surface energy of the liquid (γ_l), and the superscripts d and p represent the dispersive and the polar components of the surface energy, respectively. Young-Duprè equation, represented as

$$W_A = \gamma_l(1 + cos\theta) \quad (2)$$

, gives relationship between the solid-liquid adhesion energy (W_A), γ_l, and contact angle (θ).

Based on equations (1) and (2), θ can be rewritten as

$$\gamma_l(1 + cos\theta) = 2\sqrt{\gamma_s^d \gamma_l^d} + 2\sqrt{\gamma_s^p \gamma_l^p} \quad (3)$$

, and rearrangement of equation (3) gives:

$$\frac{\gamma_l(1+cos\theta)}{2\sqrt{\gamma_l^d}} = \sqrt{\gamma_s^p}\left(\frac{\gamma_l^p}{\gamma_l^d}\right) + \sqrt{\gamma_s^d} \quad (4)$$

where, a plot of $(\gamma_l(1 + cos\theta)/2(\gamma_l^d)^{1/2})$ as a function γ_l^p/γ_l^d gives a straight line with a slope of $(\gamma_s^p)^{1/2}$ and a y-intercept of $(\gamma_s^d)^{1/2}$ as shown below.

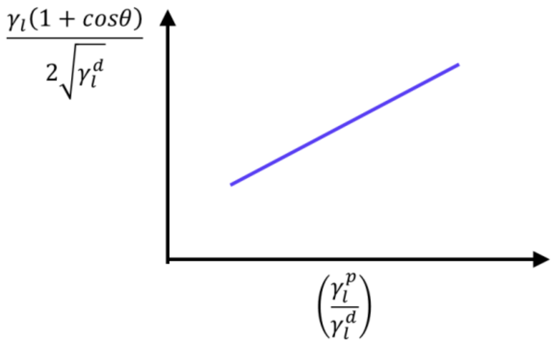

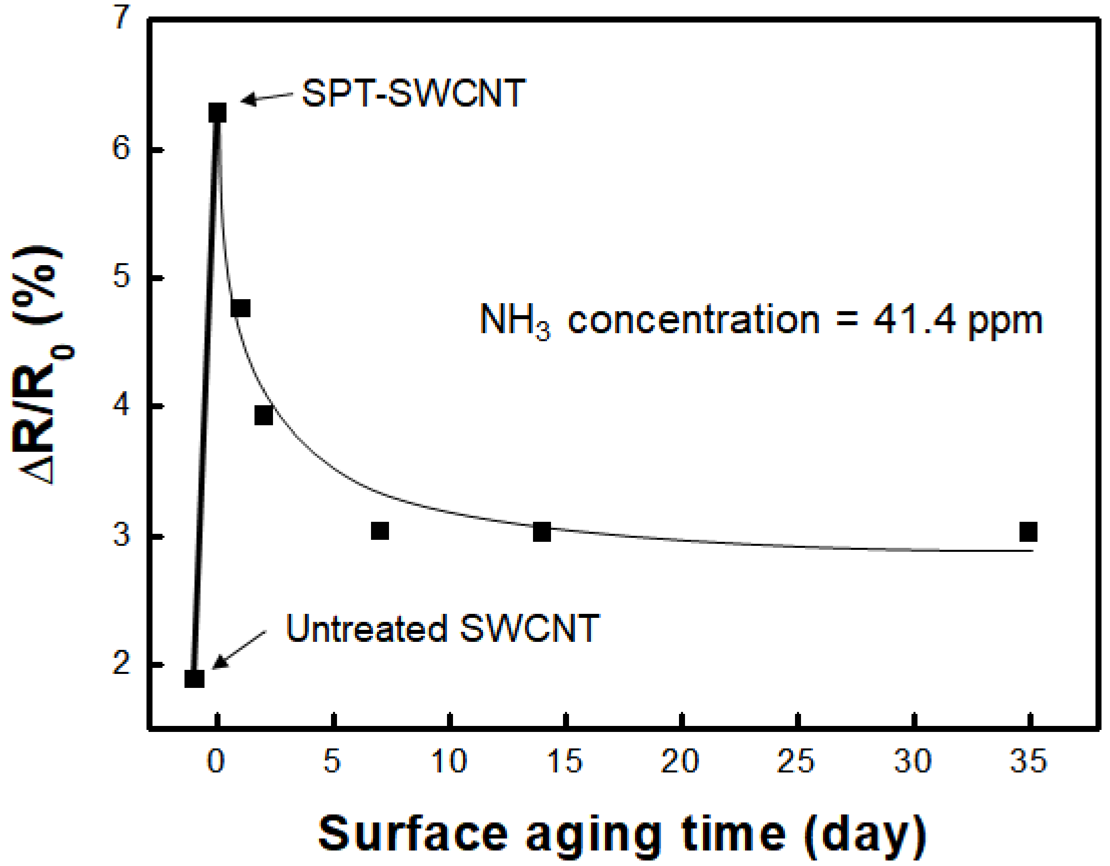

Figure S5. $\Delta R/R_0$ of the SPT-SWCNT electrodes exposed to 41.4 ppm NH_3 as a function of aging time.

Supplementary Material References

1. S.C. Ramos, G. Vasconcelos, E.F.Antunes, A.O. Lobo, V.J Trava-Airoldi, E.J. Corat, Wettability control on vertically-aligned multi-walled carbon nanotube surfaces with oxygen pulsed DC plasma and CO2 laser treatments, Diamon Relat Mater. **2010**, 19,752-755.
2. D.K Owens, R.C. Wendt, Estimation of the surface free energy of polymers, J Appl Polym Sci. **1969**, 13, 1741-1747.
